# Correlation Analysis Connects Cancer Subtypes

**DOI:** 10.1371/journal.pone.0069747

**Published:** 2013-07-08

**Authors:** Pei Lin, Zhongxi Huang

**Affiliations:** Cancer Research Institute, School of Basic Medical Sciences, Southern Medical University, Guangzhou, China; Memorial Sloan Kettering Cancer Center, United States of America

## Abstract

We provided a cross-tissue comparative analysis of between-subtype molecular commonality for ovarian cancer, breast cancer, hepatocellular carcinoma, glioma, lung squamous carcinoma and nasopharyngeal carcinoma. Our analysis showed that molecular subtypes with similar phenotype or similar clinical outcome could be correlated by their transcriptional profile and pathway profile. Pathway dysregulation across multiple cancer subtypes was also revealed by Gene Set Enrichment Analysis. Dysregulation of ‘complement and coagulation cascades’ was observed in a total of eleven subtypes across five tissues, implicating that the role of this process in personalized immune-based therapy may be worth further exploring.

## Introduction

Based on high-through genomewide transcriptome data, molecular subtypes of human cancer have been identified and characterized by different biology. For example, distinct subtypes of breast cancer were associated with different patterns of therapeutic response [1], different preferential sites of relapse [2]. In head and neck cancer, different molecular subtypes were associated with distinct patterns of copy-number alteration of canonical cancer genes [3]. In colorectal cancer, subtypes shared similarities to distinct cell types within the normal colon crypt and shows differing degrees of ‘stemness’ and Wnt signaling [4].

A recent study depicted the molecular commonality between basal-like breast cancer and ovarian cancer by correlation analysis of transcriptional profile [5], but it was unclear whether the basal-like breast cancer had a ‘friendship’ with any particular subtype of ovarian cancer. Actually, molecular cancer subtypes with similar biological characteristics were already found at different tissue sites. For example, both the Mes subtype of glioma and claudin*-*low intrinsic subtype of breast cancer were characterized by expression of mesenchymal markers and immune response [6–8]. Taking together, a question was raised that whether subtypes with similar phenotype, or similar clinical outcome, would show correlation at a molecular level? To answer this question, we performed correlation analysis of transcriptional profile and pathway profile of ovarian cancer, breast cancer, hepatocellular carcinoma (HCC), glioma, lung squamous carcinoma (lung SCC) and nasopharyngeal carcinoma (NPC). Furthermore, we analyzed pathway activities for each subtype and identified pathway frequently perturbed across different tissues.

## Materials and Methods

### Microarray dataset

All microarray datasets were downloaded from GEO. For Affymetrix data, we recalculated gene expression signal intensities by RMA [9] using Dai’s EntrezGene-center chip description file [10]. For two-color data, the normalized data matrix was used directly as provided and probes for the same gene were merged by averaging. The description of all microarray data in this study could be found in Table S1. Dataset GSEA10186 was not used for correlation analysis of transcriptional profile due to too less common genes with other datasets. Nasopharyngeal carcinoma was classified into two subtypes based on our previous work (unpublished data).

### Published Mesenchymal transition signature

Three published gene expression signature of Epithelial-Mesenchymal Transition (EMT) or Mesenchymal Transition (MT) were used in our analysis. The Taube’s ‘EMT core signature’ represent genes that shared by independent gene expression signatures (GESs) in human mammary epithelial cells (HMLE) induced to undergo an EMT by expressing Gsc, Snail, Twist, or TGF-β1 or by knocking down expression of E-cadherin [11]. The Groger’s signature included genes that were either up- or down-regulated in at least 10 independent GES of EMT [12]. The Cheng’s signature was suggested to represent a more general biological process of mesenchymal transition because it was also found in non-epithelial cancer including gliomas, neuroblastoma and Ewing’s sarcoma [13].

### Correlation analysis of transcriptional profile and pathway profile

Data was median-centered by genes in each cohort separately at first [5]. Then all gene expression values for a sample were regarded as its transcriptional profile. The adjusted datasets were submitted to GenePattern [14] for single-sample GSEA analysis [15]. The resulting pathway enrichment scores were used as the pathway profile. Positive scores indicated genes in a particular gene set are coordinately upregulated within a sample, and vice versa. Spearman rank correlation of transcriptional profile and pathway profile was calculated as the between-sample similarity metric.

### Phylogeny of cancer subtypes

The median of spearman rank correlation of transcriptional profile between two subtypes was minus by 1 and then adopted as the dissimilarity distance. Average-linkage hierarchical clustering of the resulting dissimilarity matrix was performed in R.

### Hierarchical clustering of pathway profile

Hierarchical clustering of the pathway profile matrix (rows for pathway and columns for subtype) was performed in Cluster 3.0 [16] using Spearman correlation as similarity metric and average linkage as clustering method. Scores generated by ssGSEA were used directly without any further data adjustment. The heatmap was generated in R with positive pathway enrichment scores colored by yellow while negative score colored by blue.

### Gene set enrichment analysis (GSEA)

GSEA analysis [17] was performed between tumors of a particular subtype and those of other subtypes in each cohort separately. For Affymetrix gene expression profile and the GSE10186 dataset which were represented by absolute signal intensities, signal-to-noise ratio was used as metric to calculate gene’s differential expression. For the GSE17710 dataset whose data was in log-ratio scale, difference of class means was used to calculate fold change and as metric of differential expression. KEGG pathways provided by MSigDB 3.1 were used and only pathways that significantly enriched in at least one cancer subtype at a FDR q-value < 25% were retained.

## Results

### Correlation analysis of molecular profile connected cancer subtypes

We first asked whether subtypes with similar phenotype or similar clinical outcome could be correlated by transcriptional profile and/or pathway profile. To answer this question, we measured between-subtype commonality by correlation analysis of transcriptional profile and pathway profile. A similar landscape was found for both two molecular profiles but the overall level of spearman rank correlation coefficients of pathway profiles was higher than that of transcriptional profiles (Figure S1). This may be explained by the fact that overlap of members between gene sets could add to the similarity of their statistical behavior.

**Figure 1:**
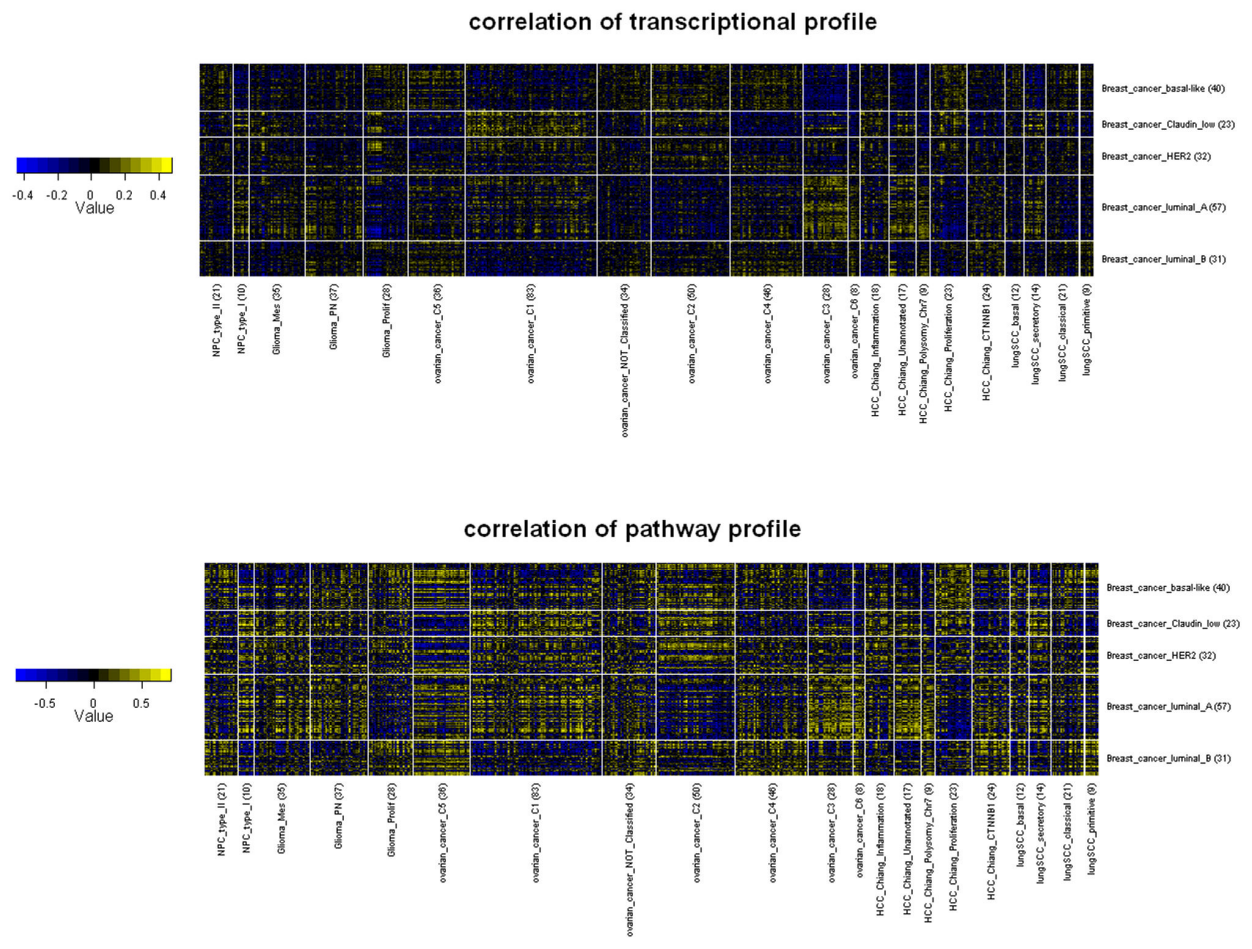
Comparison of breast cancer and other cancer. Tumor samples were grouped by subtype. Yellow grids represented positive correlation while blue grids represented negative correlation. The number of samples per subtype was inside the brackets.

Here we demonstrated molecular commonality between cancer subtypes using breast cancer as an example (Figure 1). The basal-like breast cancer tend to be positively correlated with glioma prolif, HCC proliferation, lung SCC primitive and type II NPC all of which were characterized by enhanced proliferation signature. This was consistent with the fact that basal-like breast cancer was also featured by high expression of genes associated with cell proliferation. In addition, basal-like breast cancer showed correlation with only subtypes C2, C4 and C5 of ovarian cancer [18].

Comparing with basal-like, luminal A had an almost opposite pattern of correlation. It clearly showed correlation with other better-survival subtype such as glioma PN, lung SCC secretory and ovarian cancer C3, C6. Claudin-low breast cancer was characterized by high enrichment for epithelial-to-mesenchymal transition markers and immune response genes [7]. The correlation analysis successfully captured the similarity between claudin-low and glioma Mes, which was also a mesenchymal subtype [6] with overexpression of inflammatory genes and increased density of tumor infiltrating lymphocytes [8]. Unexpectedly, an anti-correlation between claudin-low and the mesenchymal ovarian subtype C5 was observed. Heatmaps depicting between-subtype molecular commonality from the view of other cancers were also provided as supplementary figures (Figure S2-S6).

**Figure 2:**
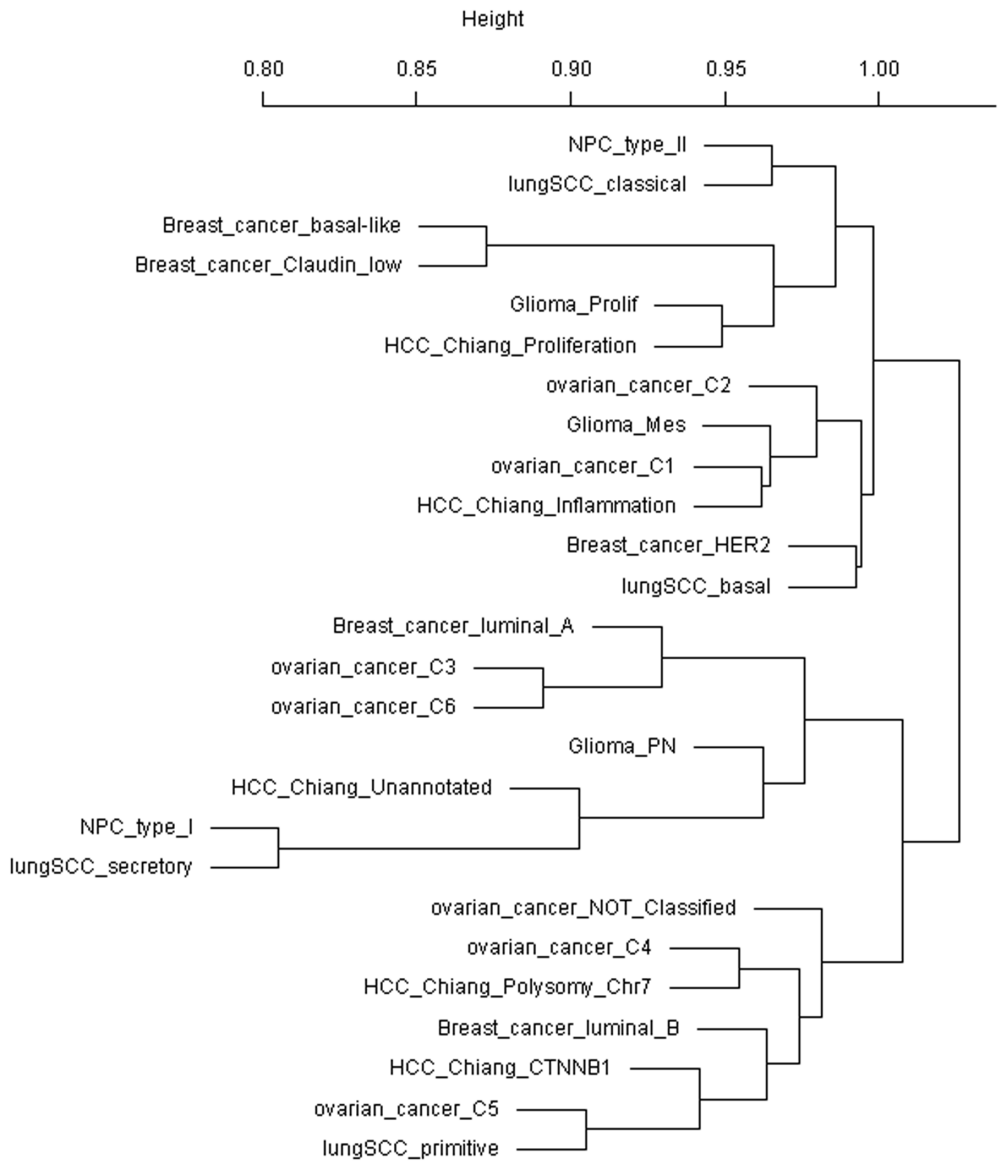
Phylogeny of cancer subtypes. The median of spearman rank correlation of transcriptional profile between two subtypes was minus by 1 and then adopted as the dissimilarity distance. Calculation of tumor phylogeny was then done by applying average linkage clustering in R.

To further gain a systematic view of between-subtype similarity, we used the median of correlation coefficients of transcriptional profiles between two subtypes as the similarity metric and calculated the phylogeny of cancer subtypes (Figure 2). We found that better-survival subtypes breast cancer luminal A and ovarian cancers C3, C6 were clustered close to glioma PN which displayed neuronal lineage markers and showed longer survival [6]. Ovarian cancer C3 and C6 represented predominantly serous low malignant potential and low-grade endometrioid subtypes [18]. Another subgroup was found which consisted of breast cancer basal-like, breast cancer claudin-low, glioma prolif and HCC Chiang’s proliferation. The former three subtypes were all with poor survival while the Chiang’s proliferation was significantly correlated with overexpression of alpha-fetoprotein (AFP) and macrovascular invasion [19]. Ovarian C1, C2 and glioma Mes were grouped with HCC Chiang’s inflammation. The former three ones were associated with higher density of tumor infiltrating lymphocytes while Chiang’s inflammation was an interferon-related subclass [19]. The phylogeny of cancer subtype also captured histology-related similarity, as subtypes of two squamous carcinoma, lung SCC and NPC, were clustered together.

### Linking cancer subtypes with pathway activities

**Figure 3:**
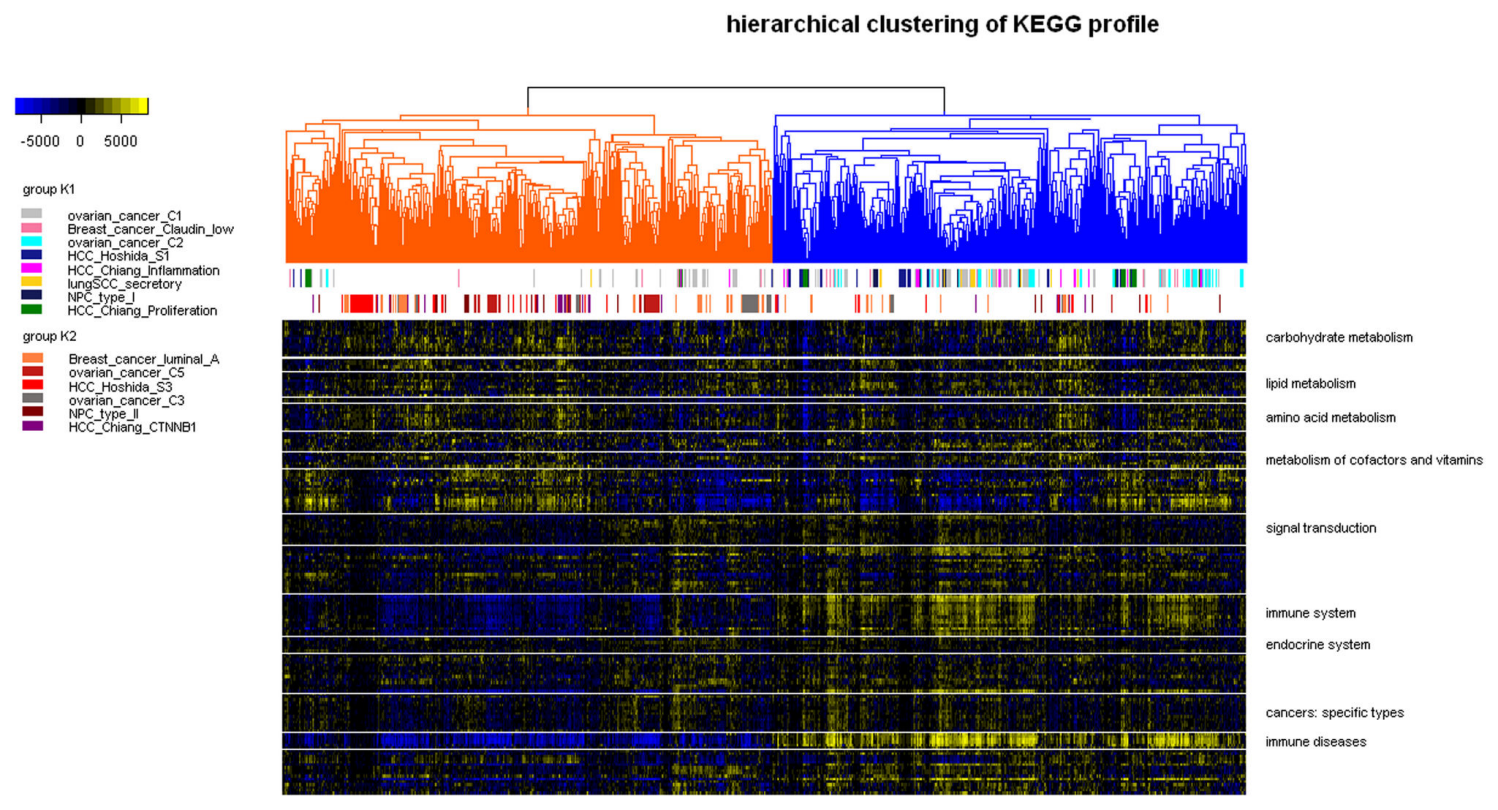
Pathway profiles across six human cancers. Each grid represents a score of pathway activity calculated by single-sample GSEA. No further adjustment of the ssGSEA score was performed. Pathways were ordered by category manually and separated from others using horizontal white lines. Only categories with more than five pathways were labeled. The dendrogram was split into two groups with group K1 colored by blue and group K2 colored by orange. The upper horizontal color bar marked subtypes overrepresented in group K1 while the lower horizontal color bar marked subtypes overrepresented in group K2. Most subtypes were significantly enriched (p-value < 0.05, Chi-squared test) except HCC Chiang’s Proliferation and CTNNB1 (p-value = 7.27e-02 and 7.86e-02, respectively).

We next sought to provide a global map of pathway activities across six types of human cancer (Figure 3). Another HCC dataset was added which was classified into three subtypes by Hoshida et al (denoted as S1, S2 and S3) [20]. Unsupervised hierarchical clustering of pathway profiles showed that samples of different cancers were mixed instead of being grouped by tissues, indicating that pathway activities in cancer were not regulated in a tissue-specific manner. It was observed that subtypes characterized by immune-related biology, including claudin-low, C1, C2, HCC Chiang’s inflammation, lung SCC secretory and type I NPC, were overrepresented in the group K1. On the other hand, luminal A, C5, S3, C3 and type II NPC were overrepresented in group K2. An obvious difference in immune-related pathway activities between these two groups was found.

We found that both Hoshida’s S1 and Chiang’s Proliferation were enriched in group K1 while Hoshida’s S3 and Chiang’s CTNNB1 were enriched in group K2. This is consistent with the observation that both Hoshida’s S1 was significantly enriched with gene signature of Chiang’s Proliferation while Hoshida’s S3 was significantly enriched with gene signature of Chiang’s CTNNB1. Thus, subtypes with similar gene expression signature could also be similar in the global landscape of pathway activities.

We also performed Gene Set Enrichment Analysis to identify pathways that associated with each subtype. As our particular interest in mesenchymal transition, published EMT signatures were also taken into analysis. At a FDR cutoff of 0.25, a total of 161 KEGG pathways were found to be significantly upregulated/downregulated in at least one subtype (Table S2). 42 pathways were only dysregulated in one tissue and may represent tissue-specific processes. For example, 14 metabolism-associated pathways were found upregulated/downregulated only in subtypes of hepatocellular carcinoma. On the other hand, 22 pathways were dysregulated in at least five tissues and therefore may represent common underlying mechanisms of carcinogenesis. ‘Complement and coagulation cascades’ was the most frequently perturbed pathway, as it was dysregulated in eleven subtypes.

GSEA of EMT signatures was consistent with the mesenchymal phenotype of claudin-low, Mes and argued that Hoshida’s S1 and ovarian cancer C1 could also be mesenchymal subtypes (Table S2). Interestingly, down-regulated arm of two EMT signatures were found significantly downregulated in C5. DAVID functional analysis showed that all the EMT signatures we used did not overlap with any immune-associated pathway (Table S3). When considering these five subtypes only, eleven pathways were downregulated only in C5 but upregulated in all other four mesenchymal subtypes. ‘Complement and coagulation cascades’ was downregulated in both C5 and Hoshida’s S1, but upregulated in C1, Mes and claudin-low.

## Discussion

In general, biological characteristics of molecular cancer subtypes could be defined by their gene expression signatures. For example, mesenchymal subtypes were usually defined by the overexpression of mesenchymal markers and underexpression of epithelial markers. Thus it may not be surprised to find common signature genes for cancer subtypes with similar biology. Instead of comparing signature genes, our study provided a systematic analysis focusing on genomewide transcriptional profile and pathway profile. Our results suggested that for subtypes characterized by similar biological characteristics, their commonality could be detected at a molecular level, indicating that the biological process alterations may play in a genomewide manner instead of only limiting to a subset of genes.

The difference between claudin-low, glioma Mes and C5 was revealed by anti-correlation of transcriptional profile and pathway profile, distinct pattern of EMT signature enrichment and opposite pattern of pathway enrichment. Such difference may be explained by the fact that C5 had strikingly low CD3+ and CD45+ cell infiltration in both tumor and stroma [18]. Immune-related processes may be relatively less involved in the acquisition of a mesenchymal trait in C5. For two potential mesenchymal subtypes, C1 was associated high stromal response and high number of stromal CD3^+^ cells [18] while S1 was characterized by TGF-beta induced Wnt activation and enrichment of an EMT-related gene set [20]. The type, location and level of tumor infiltrating lymphocytes remained unclear in Hoshida’s S1. Whether S1 and C1 were truly mesenchymal subtype required experimental validation.

‘Complement and coagulation cascades’ was an interesting process since perturbation of this pathway was observed in eleven subtypes including upregulation in C1, Mes, claudin-low and downregulation in C5, S1. Complement activation could potentially be a very important event in anti-cancer immunity and immunotherapy [21] as it could not only help in tumor clearance but also promote tumor growth [22]. Previous studies have also implicated an association between EMT and complement system. For example, C5b-9 could induce the expression of Response Gene to Complement-32 (RGC-32) which could in turn enhance metastatic phenotype by mediating TGF-β-induced EMT in human pancreatic cancer cell [23,24]. In addition, Tang Z et al showed that tubular epithelial cells exposed to complement anaphylotoxin C3a adopted phenotypic and functional characteristics of mesenchymal cells [25]. Coagulation disorders are a common problem in neoplastic patients. A hypercoagulable state could be induced by malignant cells interacting directly with hemostatic system and activating the coagulation cascade. Thrombin was formed by proteolytic cleavage of the coagulation factor II in the coagulation cascade and acted in turn as a serine protease that converts soluble fibrinogen into insoluble strands of fibrin, as well as catalyzing many other coagulation-related reactions. It was already reported that thrombin could support tumor cell malignancy [26–28]. Tumor cells could express tissue factor which consequently interacts with coagulation factor VII (FVII) and coagulation factor X (FX) to generates thrombin to enhance tumor progression [29].

In summary, the dysregulation of complement and coagulation cascades in a total of eleven subtypes across five tissues implicated that further study of this process could possibly motivate novel immunity-based strategy for personalized therapy.

## Conclusion

Our work detected molecular commonality between cancer subtypes by correlation analysis of transcriptional profile and pathway profile. Molecular classification of human cancer is just an early step towards personalized medicine. With more and more data (not limited to transcriptome) become available, we may expect more and more cancers being classified into molecular subtypes. Our method, and of course other improved and enhanced methods, could be applied to construct a more comprehensive map of cancer subtypes. With such a map, knowledge for a particular cancer subtype could provide clues for an extended understanding of its ‘friend’ subtypes in other cancers and bring potential novel therapeutic opportunities.

## Supporting Information

Figure S1 Scatter plot of correlation coefficient. A linear model was fit to the data using correlation coefficients by transcriptional profile as the independent variable. The resulting p-value and coefficient were showed.
(TIF)

Figure S2 Comparison of ovarian cancer and other cancer. Tumor samples were grouped by subtype. Yellow grids represented positive correlation while blue grids represented negative correlation. The number of samples per subtype was inside the brackets.
(TIF)

Figure S3 Comparison of glioma and other cancer. Tumor samples were grouped by subtype. Yellow grids represented positive correlation while blue grids represented negative correlation. The number of samples per subtype was inside the brackets.
(TIF)

Figure S4 Comparison of hepatocellular carcinoma and other cancer. Tumor samples were grouped by subtype. Yellow grids represented positive correlation while blue grids represented negative correlation. The number of samples per subtype was inside the brackets.
(TIF)

Figure S5 Comparison of lung squamous carcinoma and other cancer. Tumor samples were grouped by subtype. Yellow grids represented positive correlation while blue grids represented negative correlation. The number of samples per subtype was inside the brackets.
(TIF)

Figure S6 Comparison of nasopharyngeal carcinoma and other cancer. Tumor samples were grouped by subtype. Yellow grids represented positive correlation while blue grids represented negative correlation. The number of samples per subtype was inside the brackets.
(TIF)

Table S1 A brief description of all the microarray dataset used in this study. A "*" in the research type column indicated that the corresponding study defined the molecular subtypes.
(XLSX)

Table S2 GSEA results for KEGG pathways and published EMT signature. Only those with q-values less than 0.25 were included .
(XLSX)

Table S3 Results of DAVID functional analysis of published EMT signatures.
(XLSX)
