# Supplementary figures and images for: Correlation Analysis Connects Cancer Subtypes
Source: PLoS One. 2013 Jul 8;8(7):e69747. doi: 10.1371/journal.pone.0069747 (PMC3704535; doi:10.1371/journal.pone.0069747)

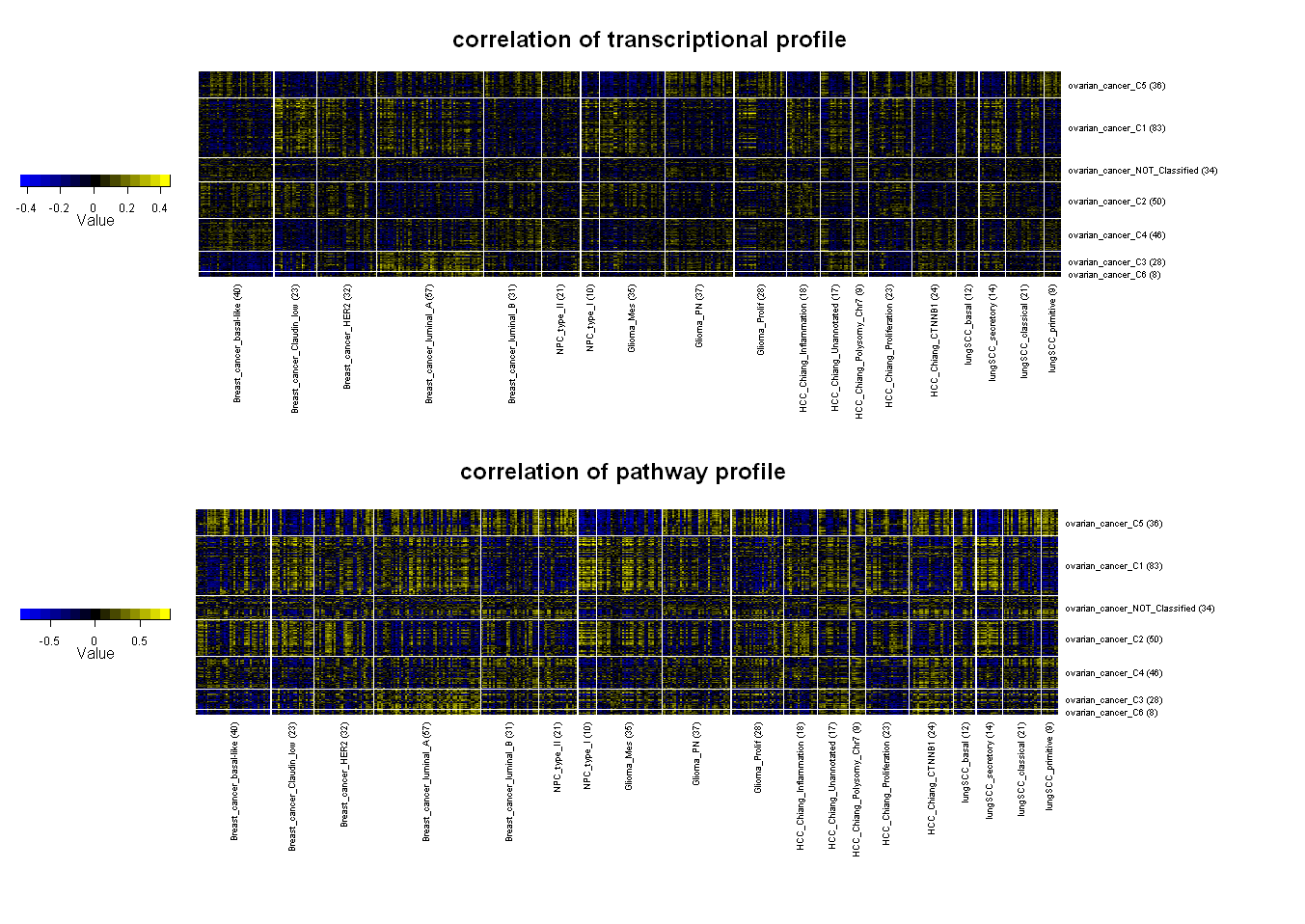

Supplement: Figure S2 — Comparison of ovarian cancer and other cancer. Tumor samples were grouped by subtype. Yellow grids represented positive correlation while blue grids represented negative correlation. The number of samples per subtype was inside the brackets. (TIF) [file pone.0069747.s002.tif]

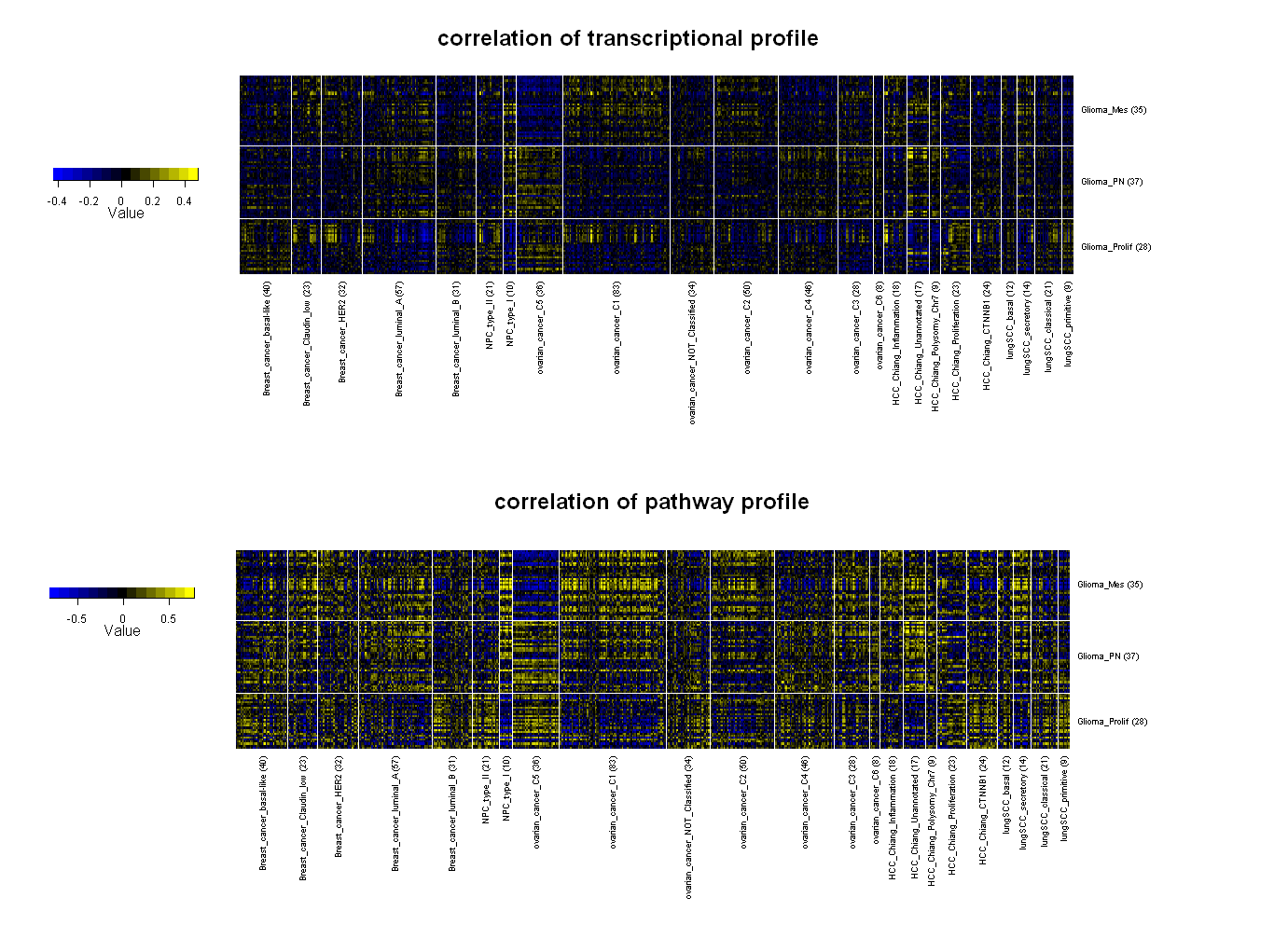

Supplement: Figure S3 — Comparison of glioma and other cancer. Tumor samples were grouped by subtype. Yellow grids represented positive correlation while blue grids represented negative correlation. The number of samples per subtype was inside the brackets. (TIF) [file pone.0069747.s003.tif]

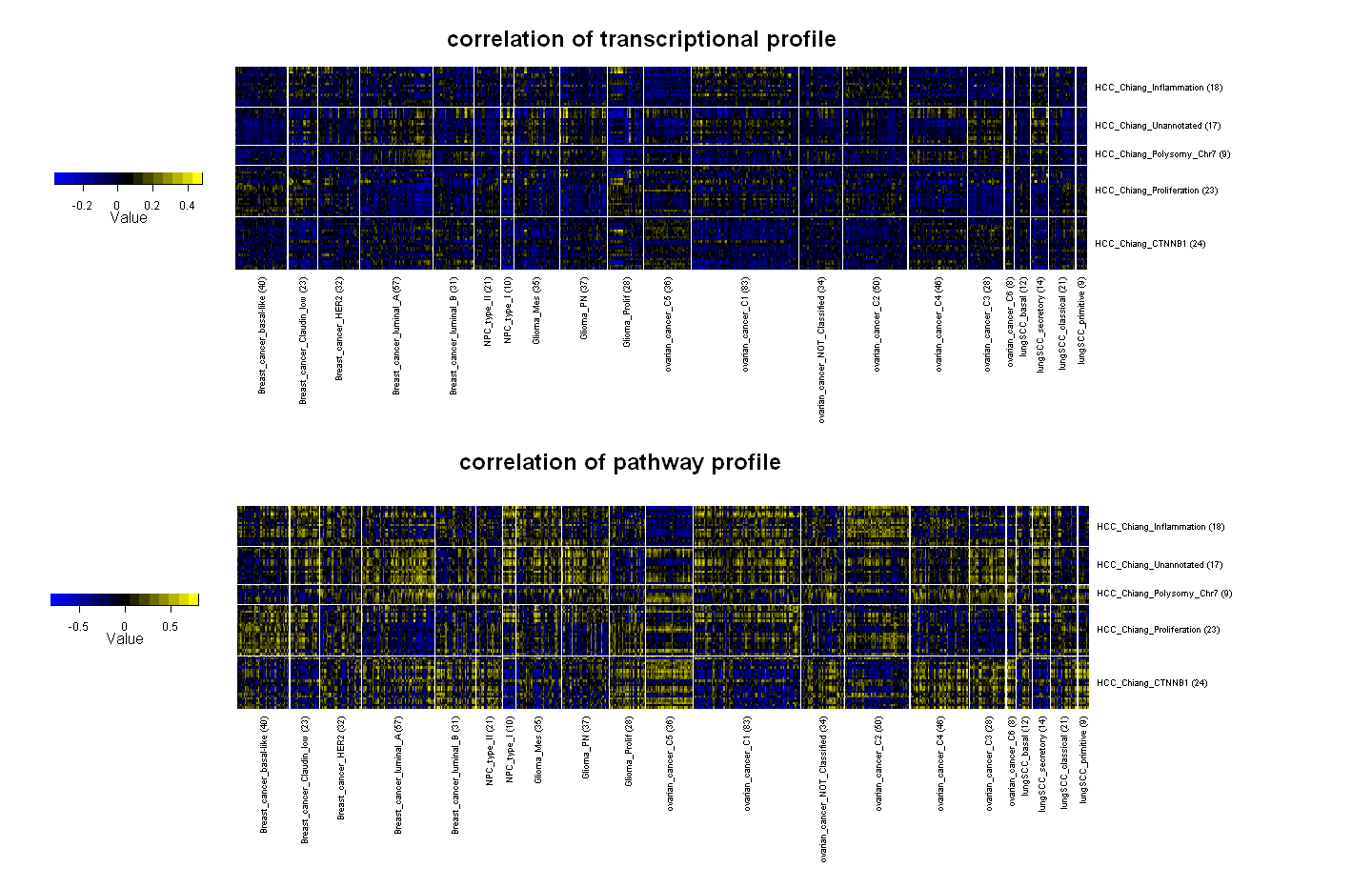

Supplement: Figure S4 — Comparison of hepatocellular carcinoma and other cancer. Tumor samples were grouped by subtype. Yellow grids represented positive correlation while blue grids represented negative correlation. The number of samples per subtype was inside the brackets. (TIF) [file pone.0069747.s004.tif]

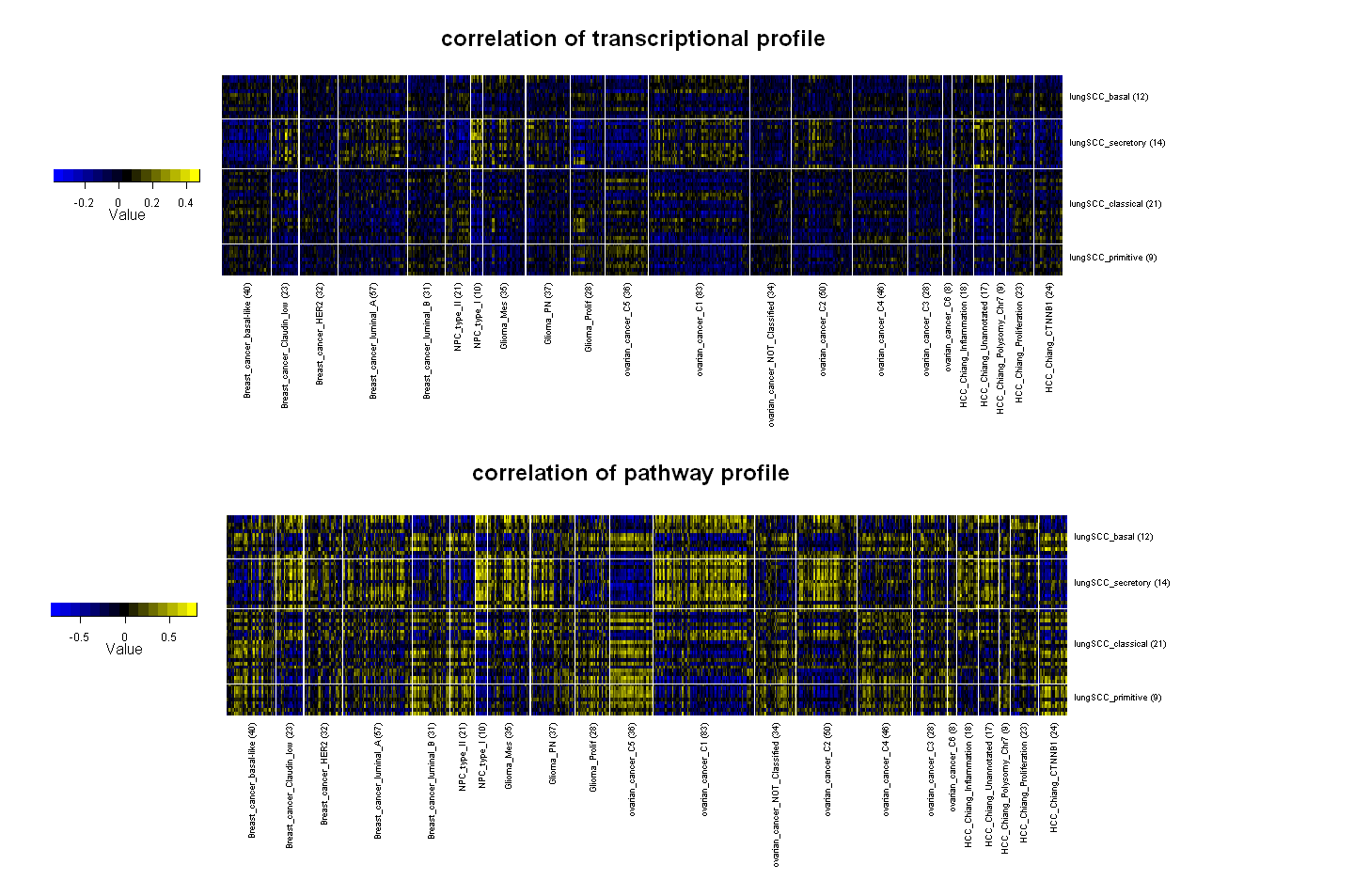

Supplement: Figure S5 — Comparison of lung squamous carcinoma and other cancer. Tumor samples were grouped by subtype. Yellow grids represented positive correlation while blue grids represented negative correlation. The number of samples per subtype was inside the brackets. (TIF) [file pone.0069747.s005.tif]

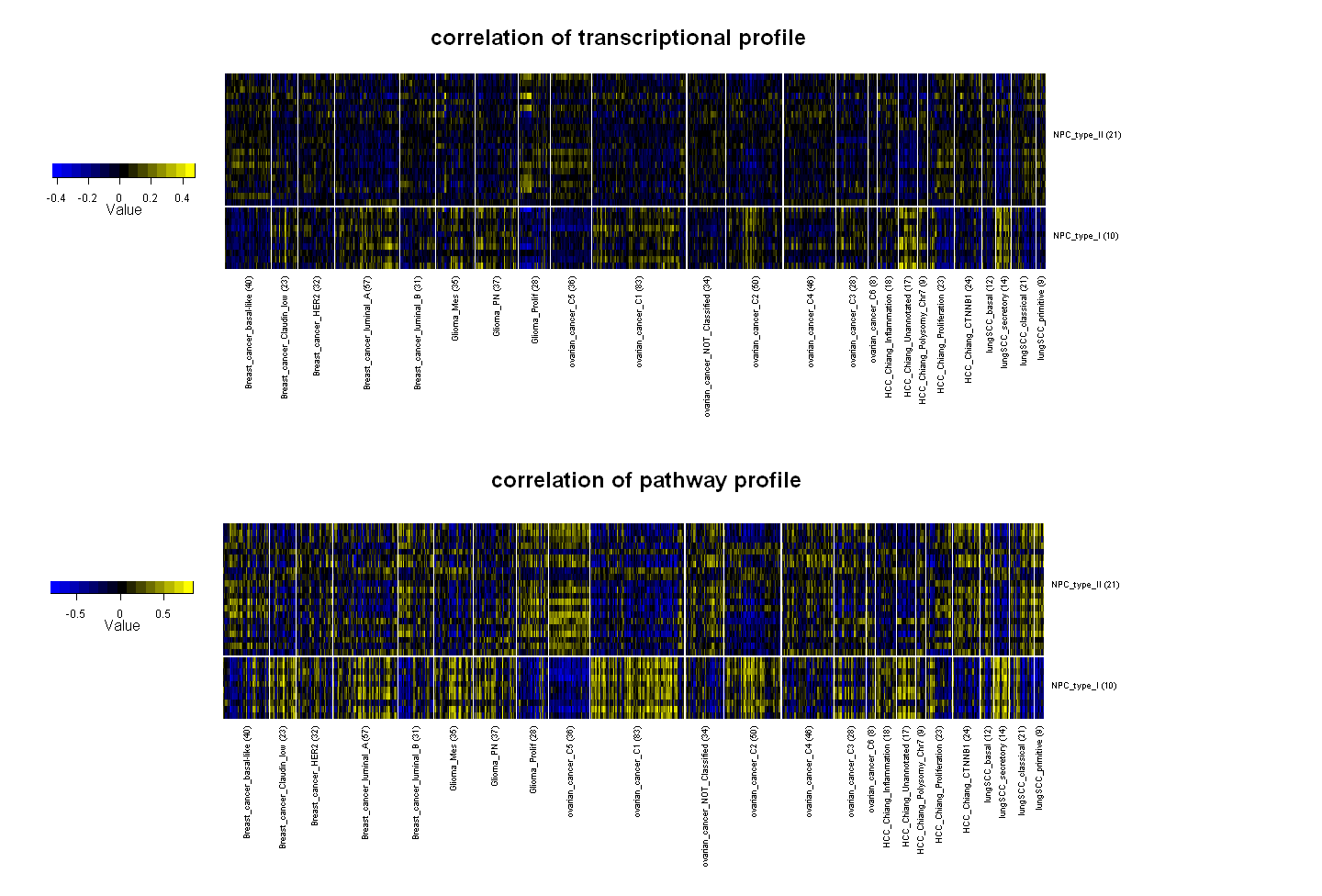

Supplement: Figure S6 — Comparison of nasopharyngeal carcinoma and other cancer. Tumor samples were grouped by subtype. Yellow grids represented positive correlation while blue grids represented negative correlation. The number of samples per subtype was inside the brackets. (TIF) [file pone.0069747.s006.tif]
